# Supplementary material for: Genome-wide association study of biological nitrogen fixation traits in mini-core cowpea germplasm
Source: PLoS One. 2025 May 9;20(5):e0322203. doi: 10.1371/journal.pone.0322203 (PMC12063810; doi:10.1371/journal.pone.0322203)
Supplement: Attachment — Submitted filename: Reviewrer comments.pdf [file pone.0322203.s006.pdf]

To, Date: 17/01/2025

The editor
PLOS ONE
plosone@plos.org

The manuscript is titled "**Genome-Wide Association Study of Biological Nitrogen Fixation-Related Traits in a Mini-core Subset of Cowpea Germplasm"** The work focuses on an essential part of sustainable agriculture: boosting BNF efficiency in cowpea, which helps to increase crop yields while also restoring soil fertility. The study is especially pertinent in Sub-Saharan Africa, where food security and soil health are critical issues. This paper contributes significantly to the subject of legume genetics and sustainable agriculture. It gives useful information about the genetic architecture of BNF in cowpea, with practical implications for breeding efforts. I recommend that this work be accepted with minor adjustments, as noted below.

Comments for improvement

• Line Numbers 25 to 47, The abstract is comprehensive; however, it could be streamlined by emphasizing the key findings. For instance, one could minimize the discussion of methods and highlight significant results, such as the identified genomic regions. Emphasize the practical implications of your findings, particularly how these genomic insights can be directly utilized in cowpea breeding programs.

• Line numbers 62 to 79, The introduction discusses BNF efficiency; however, a clear comparison with earlier GWAS studies on legumes would enhance the rationale.

• Line numbers 133-134, Nodule formation in legumes is significantly affected by the rhizobial strain, as the compatibility between the legume plant and specific rhizobial strains directly influences nodule efficiency. What was the rationale for selecting a single rhizobial strain in your study, given the availability of multiple strains?

•Indicate if an independent dataset or validation population was utilized to verify the identified significant SNPs. Consider including this as a limitation if applicable.

•Present a justification for the utilization of the mrMLM.GUI package and elucidate the selection of six distinct methods.

• Line numbers 490 to 493, Elaborate on the functional importance of the identified candidate genes. How do these genes interact with other traits or processes that influence cowpea productivity?

•Several points are repeated in the discussion, including the function of WRKY genes and the necessity for KASP markers. Enhancing the flow and reducing redundancy can be achieved by streamlining these points.

•The references to supporting studies are significant; however, their integration into the narrative could be improved. Instead of enumerating genes identified in other species, directly compare their functions across species and their significance to cowpea. In the discussion, the association between the SNP markers and their corresponding genes should be articulated more clearly, avoiding redundancy in points related to various traits (e.g., WRKY and Thoumatin genes).

Sincerely

Basavaraja, T

Scientist, ICAR-IIOR, Hyderbad
