# Supplementary material for: Genome-wide association study of biological nitrogen fixation traits in mini-core cowpea germplasm
Source: PLoS One. 2025 May 9;20(5):e0322203. doi: 10.1371/journal.pone.0322203 (PMC12063810; doi:10.1371/journal.pone.0322203)
Supplement: Attachment — Submitted filename: Response to Reviewer #2.docx [file pone.0322203.s007.docx]

Dear Reviewer #2,

We appreciate your thoughtful comments, suggestions, and questions regarding our manuscript. Below are our responses to each of your points

**Reviewer comment 1:**

Line Numbers 25 to 47, The abstract is comprehensive; however, it could be streamlined by emphasizing the key findings. For instance, one could minimize the discussion of methods and highlight significant results, such as the identified genomic regions. Emphasize the practical implications of your findings, particularly how these genomic insights can be directly utilized in cowpea breeding programs.

**Authors response 1:**

We have revised the abstract and focused more on the key findings, emphasizing identified significant SNP markers (genomic regions), candidate genes and their implications in cowpea breeding rather than a detailed discussion of methods (Line 32-61 in the manuscript with track changes).

**Reviewer comment 2:**

Line numbers 62 to 79, The introduction discusses BNF efficiency; however, a clear comparison with earlier GWAS studies on legumes would enhance the rationale

**Authors response 2:**

The introduction has been updated to include a clearer comparison with previous GWAS studies on legumes. (Line 118-132)

**Reviewer comment 3:**

Line numbers 133-134, Nodule formation in legumes is significantly affected by the rhizobial strain, as the compatibility between the legume plant and specific rhizobial strains directly influences nodule efficiency. What was the rationale for selecting a single rhizobial strain in your study, given the availability of multiple strains?

**Authors response 3:**

We selected a single rhizobial strain to ensure consistency in nodulation responses across all genotypes. We used *Bradyrhizobium spp* which was reported by previous studies (Somasegaran and Hoben, 2012) as a cowpea-type strain and the most common rhizobial strain found in approximately 70.3% of cowpea-growing soils in Africa (Abaidoo et al., 2000); and as the identified genotypes can be recommended for multilocational trials. A statement highlighting the choice of this particular strain has been added (line 175-177).

**Reviewer comment 4:**

Indicate if an independent dataset or validation population was utilized to verify the identified significant SNPs. Consider including this as a limitation if applicable.

**Authors response 4:**

No independent validation population was used in this study. We had explicitly mentioned this limitation and suggested future studies incorporating validation datasets for further confirmation (line 722-728).

**Reviewer comment 5:**

Present a justification for the utilization of the mrMLM.GUI package and elucidate the selection of six distinct methods.

**Authors response 5:**

The choice of the mrMLM.GUI package with its six integrated methods was based on its efficiency in detecting loci with small effects in multi-locus GWAS. Hence, mrMLM.GUI is better than other multi-locus models such GAPIT, TASSEL and FamCPU due to its (i) multi-locus detection, (ii) GUI-based user interface, (iii) very high power for polygenic traits, (iv) low false positive rate and (v) advanced environmental covariate (da Silva Linge et al., 2021; Zhang et al., 2020). We have added this statement and therefore improved the explanation in the manuscript (line 252-257).

**Reviewer comment 6:**

Line numbers 490 to 493, Elaborate on the functional importance of the identified candidate genes. How do these genes interact with other traits or processes that influence cowpea productivity?

**Authors response 6:**

We have expanded on how the identified genes interact with other traits influencing cowpea productivity, providing a broader context for their functional roles (Line 669-670, 671-674, 697-701).

**Reviewer comment 7:**

Several points are repeated in the discussion, including the function of WRKY genes and the necessity for KASP markers. Enhancing the flow and reducing redundancy can be achieved by streamlining these points.

**Authors response 7:**

We have revised the discussion to improve the flow and eliminate redundancy regarding the WRKY gene function. Specifically, we streamlined the description of WRKY genes across different genomic regions, ensuring that their role is clearly stated without unnecessary repetition. Additionally, we have refined the discussion on genomic markers to maintain clarity and coherence (Line 663-678).

**Reviewer comment 8:**

The references to supporting studies are significant; however, their integration into the narrative could be improved. Instead of enumerating genes identified in other species, directly compare their functions across species and their significance to cowpea. In the discussion, the association between the SNP markers and their corresponding genes should be articulated more clearly, avoiding redundancy in points related to various traits (e.g., WRKY and Thoumatin genes).

**Authors response 8:**

We have revised the discussion to improve the integration of supporting studies, ensuring a clearer comparison of gene functions across species and their relevance to cowpea. Additionally, we have articulated the association between SNP markers and their corresponding genes more explicitly while eliminating redundancy (line 663-678 for WRKY and 693-705 for Thoumatin).

In addition, some changes occurred during this revision by co-authors. We noticed that the 3 SNP tables were long and unnecessary in the main manuscript yet the only interesting SNPs are those depicted by more than one multi-locus GWAS model for each trait. Therefore, for easy reading and comprehension, we summarized the tables in one and put the full tables in supplementary materials. Note that the first SNP (Vu6_23154812) with related gene Vigun06g101100 (ACBP genes) has been removed in the revised version because, it was depicted by only one model, even though it was associated with the number of nodules per plant (NN). Thus, the whole sub-section **“Genome-wide association** **signals” (**which was also modified**)** under the **“Results”** section has been modified accordingly. Consequently, the tables on gene annotation (Table 2) and the one on the profile of alleles at the top 15 genotypes (table 3) have been also modified and all the information in the discussion section related to ACBP gene has been removed **(line 636-643).**

Lastly, the SNP marker names were wrongly reported. They were confused with their position on respective chromosomes instead using their official names (SNP ID) as reported in other scientific studies on cowpea. The changes can be seen in Table 2 for example and throughout the manuscript.

We hope these responses clarify your concerns and appreciate your valuable feedback

Sincerely

Gelase Nkurunziza

Submitting author

March 11^th^ 2025

References

1.da Silva Linge, C., Cai, L., Fu, W., Clark, J., Worthington, M., Rawandoozi, Z., Byrne, D. H., & Gasic, K. (2021). Multi-Locus Genome-Wide Association Studies Reveal Fruit Quality Hotspots in Peach Genome. *Frontiers in Plant Science*, *12*. <https://doi.org/10.3389/fpls.2021.644799>

2.Somasegaran, P., & Hoben, H. J. (2012). *Handbook for rhizobia: methods in legume-Rhizobium technology* (Springer). Springer-Verlag New York.
